# NK3R signalling in the posterodorsal medial amygdala is involved in stress‐induced suppression of pulsatile LH secretion in female mice

**DOI:** 10.1111/jne.13384

**Published:** 2024-03-22

**Authors:** Deyana Ivanova, Margaritis Voliotis, Krasimira Tsaneva‐Atanasova, Kevin T. O'Byrne, Xiao‐Feng Li

**Affiliations:** ^1^ Department of Women and Children's Health, Faculty of Life Science and Medicine King's College London London UK; ^2^ Department of Medicine, Division of Endocrinology, Diabetes and Hypertension, Brigham and Women’s Hospital Harvard Medical School Boston MA USA; ^3^ Department of Mathematics and Living Systems Institute, College of Engineering, Mathematics and Physical Sciences University of Exeter Exeter UK

**Keywords:** LH pulsatility, MePD, NK3R, stress

## Abstract

Psychosocial stress negatively impacts reproductive function by inhibiting pulsatile luteinizing hormone (LH) secretion. The posterodorsal medial amygdala (MePD) is responsible in part for processing stress and modulating the reproductive axis. Activation of the neurokinin 3 receptor (NK3R) suppresses the gonadotropin‐releasing hormone (GnRH) pulse generator, under hypoestrogenic conditions, and NK3R activity in the amygdala has been documented to play a role in stress and anxiety. We investigate whether NK3R activation in the MePD is involved in mediating the inhibitory effect of psychosocial stress on LH pulsatility in ovariectomised female mice. First, we administered senktide, an NK3R agonist, into the MePD and monitored the effect on pulsatile LH secretion. We then delivered SB222200, a selective NK3R antagonist, intra‐MePD in the presence of predator odour, 2,4,5‐trimethylthiazole (TMT) and examined the effect on LH pulses. Senktide administration into the MePD dose‐dependently suppresses pulsatile LH secretion. Moreover, NK3R signalling in the MePD mediates TMT‐induced suppression of the GnRH pulse generator, which we verified using a mathematical model. The model verifies our experimental findings: (i) predator odour exposure inhibits LH pulses, (ii) activation of NK3R in the MePD inhibits LH pulses and (iii) NK3R antagonism in the MePD blocks stressor‐induced inhibition of LH pulse frequency in the absence of ovarian steroids. These results demonstrate for the first time that NK3R neurons in the MePD mediate psychosocial stress‐induced suppression of the GnRH pulse generator.

## 1 INTRODUCTION

Psychosocial stress exerts a profound inhibitory effect on reproduction in mammals, including humans.^1^ Predator odour exposure delays puberty^2^ and suppresses pulsatile^3^, ^4^ and surge luteinizing hormone (LH) secretion^5^ in rodents. Hypothalamic arcuate nucleus (ARC) neurons co‐expressing kisspeptin (kiss1), neurokinin B (NKB) and dynorphin, also known as KNDy neurons, are a critical component of the gonadotropin‐releasing hormone (GnRH) pulse generator^6^, ^7^, ^8^ controlling the hypothalamic–pituitary–gonadal (HPG) axis. The synchronous activity of the KNDy neuronal network leads to a robust activation of GnRH neurons inducing pulsatile GnRH and LH release.^9^, ^10^ Acute exposure to psychosocial stressors reduces c‐fos expression in ARC KNDy neurons in mice^11^ while suppressing pulsatile LH secretion.^12^ The neural circuits engaged to mediate the effect of psychological stress on the GnRH pulse generator remain to be fully characterised.

The amygdala processes threatening stimuli and is a key part of the limbic brain neurocircuitry responsible for integrating anxiety and fear states with the HPG and hypothalamic pituitary adrenal (HPA) axes. The posterodorsal sub‐nucleus of the medial amygdala (MePD) is an upstream modulator of LH pulsatility^3^, ^12^, ^13^ and pubertal timing in mice,^14^ rats^15^ and monkeys.^16^ Psychosocial stress elevates c‐fos expression in the MePD^17^ while lesioning of this region blocks restraint stress‐induced suppression of LH pulsatility in rats.^3^ The MePD is robustly activated in response to predator odour, a psychosocial stressor.^18^ We have recently shown that chemogenetic‐mediated inhibition of MePD Urocortin3 neurons in the presence of 2,4,5‐trimethylthiazole (TMT), a predator odour, attenuates the stress‐induced suppression of LH pulses and rise in corticosterone (CORT) secretion in mice.^12^ The MePD projects to ARC KNDy neurons in rodents,^19^, ^20^ thus it may be a central hub involved in integrating external stress signals with the GnRH pulse generator.

NKB is a highly specific endogenous ligand for the neurokinin 3 receptor (NK3R), a member of the tachykinin (*Tac*) receptor family.^21^ Hypothalamic arcuate nuclear NK3R and NKB neurocircuits are indispensable for the control and maintenance of reproductive processes.^8^, ^10^, ^22^, ^23^ Inactivating mutations in *Tac3* or *TacR3* genes, encoding NKB and its receptor, respectively, have been demonstrated in patients suffering hypogonadotropic hypogonadism.^24^ NK3R is also expressed in the amygdala in mice, rats, monkeys and humans.^25^, ^26^, ^27^ Recently, specific NKB signalling in the MePD has been shown to have a stimulatory influence on the secretion of LH in the presence of oestrogen.^28^ The NK3R and NKB system in the amygdala has also been shown to play a role in stress, anxiety and mood disorders.^29^, ^30^, ^31^ In post‐traumatic stress disorder (PTSD) mice models, stress‐induced elevations of the *Tac2* gene expression, encoding NKB, occur in the central nucleus of the amygdala.^32^ Administration of SB222200, a NK3R antagonist, via an intracerebroventricular (ICV) route blocks the inhibitory effect of stress on pulsatile LH secretion in ovariectomised (OVX) rats.^29^ Therefore, we hypothesise that NKB and NK3R signalling in the MePD may be involved in mediating the effects of psychosocial stress on GnRH pulse generator activity.

In this study, we aimed to determine whether signalling through the NK3R in the MePD mediates the suppressive effect of acute predator odour exposure on LH pulsatility in adult OVX mice. First, we determined the effect of intra‐MePD administration of senktide, an NK3R agonist, on pulsatile LH secretion. We then investigated whether intra‐MePD administration of the highly selective NK3R antagonist, SB222200, would block predator odour stress‐induced inhibition of pulsatile LH secretion. In addition, we used mathematical modelling to verify the potential interactions between stress and NK3R signalling in the MePD regulating GnRH pulse generator frequency.

## 2 MATERIALS AND METHODS

### 2.1 Mice

Female C57Bl6/J mice aged between 6 and 8 weeks and weighing between 19 and 23 g were purchased from Charles River Laboratories International, Inc. Mice were kept in individually ventilated cages equipped with wood‐chip bedding and nesting material, and food and water ad libitum and sealed with a HEPA‐filter at 25 ± 1°C in a 12:12 h light/dark cycle, lights on at 07:00 h. All procedures were carried out following the United Kingdom Home Office Regulations and approved by the Animal Welfare and Ethical Review Body Committee at King's College London.

### 2.2 Stereotaxic cannula implantation

Surgical procedures were carried out on animals under general anaesthesia. This was achieved using Ketamine (Vetalar, 100 mg/kg, i.p.; Pfizer, Sandwich, UK) and xylazine (Rompun, 10 mg/kg, i.p.; Bayer, Leverkusen, Germany). Mice were bilaterally OVX while secured in the Kopf stereotaxic frame (Kopf Instruments, Model 900) just before cannula implantation surgery was performed. Mice aged between 8 and 10 weeks were used for these experiments. Unilateral or bilateral cannula implantation was performed in the MePD using the robot stereotaxic system (Neurostar, Tubingen, Germany). Target coordinates, 2.30 mm lateral, −1.55 mm from bregma, at a depth of −4.94 mm below the skull surface, were used for the MePD, obtained with the mouse brain atlas of Paxinos and Franklin.^33^ The skull was revealed by making a midline incision in the scalp and a small hole was drilled in the skull above the MePD. Unilateral or bilateral 26‐gauge guide cannula (Plastics One, Roanoke, VA, USA) were implanted reaching the MePD. The guide cannula was secured on the skull using dental cement (Super‐Bond Universal Kit, Prestige Dental, UK). Sutures were used to close incisions made in the skin. Mice were left to recover for 1‐week post‐surgery. Following the 1‐week recovery period, mice were handled daily to acclimatise them to experimental procedures for a further 2 weeks.

### 2.3 Senktide administration into the MePD and blood sampling for measurement of LH pulses

To test the effects of intra‐MePD senktide (Tocris, UK) administration on pulsatile LH secretion, OVX mice implanted with a unilateral cannula on the right side were subjected to tail‐tip blood sampling, as described previously.^34^ Administration of senktide and blood sampling was performed between 09:00 and 13:00 h. During blood sample collection, 5 μL blood was collected for 2 h every 5 min. Unilateral internal cannula (Plastics One) attached to extension tubes (0.58 mm ID, 0.96 mm OD) was preloaded with senktide or artificial cerebrospinal fluid (aCSF) as vehicle control was fitted into the guide cannula. To reach the MePD, the internal cannula extends 0.5 mm from the tip of the guide cannula. The distal ends of the tubing, extending beyond the cage, were attached to 5 μL Hamilton syringes (Waters Ltd, Elstress, UK) fitted into a PHD 2000 Programmable syringe pump (Harvard Apparatus, MA, USA) for continuous infusion of the drug at a constant rate. The mice were free to move inside the cage with food and water for the entirety of the experiment. After 1 h of control blood sampling, mice were given an initial bolus injection of senktide (0.01, 0.1 or 1 pmol in 0.20 μL at a rate of 0.04 μL/min) over 5 min, followed by a continuous infusion (0.02, 0.2 or 2 pmol in 0.80 μL delivered at a rate of 0.015 μL/min) for the remaining 55 min of blood sampling. The doses of senktide administered are much smaller than those previously reported for intra‐MePD senktide administration in mice.^28^ Intra‐MePD senktide infusions were performed between 60 and 120 min of the total blood sampling period and administration of aCSF was performed in the same manner for controls. The mice received the three doses of senktide and aCSF in a random order, with at least 2 days between experiments.

### 2.4 SB222200 delivery into the MePD during predator odour‐exposure and blood sampling for LH pulse measurement

We tested whether SB222200, a selective NK3R antagonist, administration into the MePD can block the suppressive effect of TMT on LH pulsatility. SB222200 was delivered into both sides of the MePD in OVX mice implanted with a bilateral cannula while mice were acutely exposed to TMT (synthetic extract of fox urine; Sigma‐Aldrich, UK) during blood sampling. The dose of SB222200 administered is smaller than those previously reported for intra‐nuclear SB222200 administration in mice.^8^, ^35^ After a 50 min control blood sampling period, mice were given an initial bolus dose of 4.20 pmol SB222200 in 0.40 μL delivered at a rate of 0.08 μL/min over 5 min (i.e., 0.20 μL to each MePD sub‐nucleus), followed by a continuous infusion of 8.30 pmol SB222200 in 1.00 μL at a rate of 0.015 μL/min (i.e., 0.50 μL to each MePD sub‐nucleus) for the remaining 65 min of experimentation. At 60 min, mice were exposed to TMT for the remaining 1 h duration of the experiment, as previously described.^12^ Briefly, 12 μL of TMT (≥98% purity) was pipetted on a small circular piece of filter paper and placed in a petri dish in the centre of the cage, with blood sampling continued for the remainder of the experiment. Controls were given an empty petri dish in the centre of the cage and were kept in a separate room to avoid TMT exposure. The administration of SB222200 only was performed in the same manner excluding TMT exposure, as control. The mice received all treatments in a random order, as described above.

### 2.5 Validation of cannula implant site

A lethal dose of ketamine was used for the termination of the mice once experimental procedures were completed. The mice were subject to transcardial perfusion with heparinised saline for 5 min followed by ice‐cold 4% paraformaldehyde (PFA) in phosphate buffer (pH 7.4) for 15 min using a pump (Minipuls, Gilson, Villiers Le Bel, France). After the perfusion, the brains were collected immediately and fixed in 15% sucrose in 4% PFA at 4°C and left to sink to the bottom of the tube. Once sunk, they were transferred to 30% sucrose in phosphate‐buffered saline left to sink to the bottom of the tube. The brains were then dried from the solution and snap frozen in isopropanol on dry ice and stored at −80°C. Every third coronal brain section was collected, 30‐μm/section, using a cryostat (Bright Instrument Co., Luton, UK) from −1.34 to −2.70 mm from bregma was collected, corresponding to the MePD region. The sections were placed on microscope slides, air dried and covered with ProLong Antifade mounting medium (Molecular Probes, Inc., OR, USA). Using Axioskop 2 Plus microscope equipped with Axiovision, version 4.7 (Zeiss) the cannula position was examined and only data from animals with correct cannula placement in the MePD were analysed.

### 2.6 LH pulse detection and analysis

To measure the LH concentration in the blood samples collected, they were processed with an LH ELISA assay, as described by Steyn et al.^36^ The capture antibody (monoclonal antibody, anti‐bovine LHβ subunit, AB_2665514; dilution 1:1000) was purchased from Department of Animal Science at the University of California, Davis. The mouse LH standard (AFP‐5306A; dilution 1:25) and primary antibody (polyclonal antibody, rabbit LH antiserum, AB_2665533; dilution 1:40) were obtained from Harbor‐UCLA. The secondary antibody (horseradish peroxidase‐linked donkey anti‐rabbit IgG polyclonal antibody, AB_772206; no dilution) was purchased from VWR International (Leicestershire, UK). The inter‐assay and intra‐assay variations were 10.2% and 4.6%, respectively, and the assay sensitivity was 0.0015 ng/mL. To analyse the results from the LH ELISA assay, the optical density's of the standards were plotted against the log of the standard concentrations. Non‐linear regression was used to fit the points and various parameters were extracted to calculate the concentration of LH (ng/mL) in the blood, as previously described.^36^ The concentration of LH at every time point of blood collection was plotted as a line and scatter graph using Igor Pro 7, Wavemetrics, Lake Oswego, OR, USA. LH pulses were detected using the DynPeak algorithm.^37^ To examine the effect of the various treatments on pulsatile LH secretion the mean LH inter‐pulse interval (IPI) was determined by measuring the time interval between the peak of one pulse and the peak of the subsequent pulse. On occasions where there were no LH pulses observed during the post‐treatment period, the IPI was given a value from the peak of the LH pulse immediately before the beginning of treatment to the end of blood sample collection. When a peak is identified at time 60 min, this point is included in the IPI interval for the post‐treatment period.

### 2.7 Statistics

Senktide, aCSF, TMT and SB222200 treated female C57Bl6/J mice were compared between groups using repeated measures two‐way analysis of variance (ANOVA) with a Tukey post‐hoc. Statistics were performed using Igor Pro 7, Wavemetrics, Lake Oswego, OR, USA and R version 4.3.1, R package rstatix, Type 3, Richmond Hill, ON, Canada. Data were represented as mean ± SEM and +*p* < .05, ++*p* < .001 and +++*p* < .0001 were considered to be significant.

### 2.8 Mathematical model

We have already extended a previously published mathematical model of the ARC KNDy neuronal population incorporating inputs from the MePD neural circuitry comprised of kiss1, GABA and glutamate signalling.^8^, ^10^, ^38^ In the current study, we build upon this existing model to test our hypothesis regarding the interaction between stress and NK3R signalling in the MePD. The focus of this model was to test how NK3R activation in the MePD could affect pulsatile LH secretion and whether MePD NK3R signalling is involved in mediating stress‐induced inhibition of the GnRH pulse generator. The current model incorporates (i) the disinhibitory GABA‐GABA pathway from the MePD, (ii) glutamatergic projections from the MePD onto the ARC and (iii) NK3R expression in the MePD kiss1 population. The model was implemented and run in MATLAB. The model along with the code for reproducing the simulations presented here can be downloaded from https://git.exeter.ac.uk/mv286/kndy-model-with-mepd-inputs.

## 3 RESULTS

### 3.1 Validation of cannula placement

**FIGURE 1:**
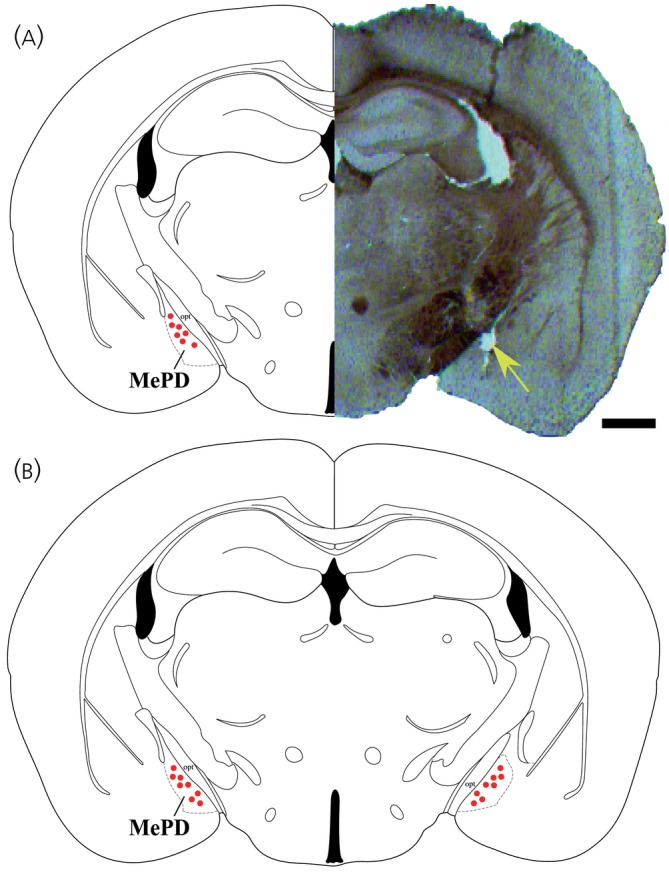
Coronal section showing correct cannula placement in the posterodorsal medial amygdala (MePD). (A) A representative example of a coronal brain section from one mouse with a unilateral cannula implant aligned to the equivalent schematic mouse brain stereotaxic coordinates and red dots in the MePD indicate the correct position of unilateral cannula placement. (B) Schematic mouse brain stereotaxic coordinates and red dots in the MePD indicate the correct position of bilateral cannula placement. Scale bars represent (A) 500 μm. Yellow arrow points to cannula implant site in MePD.

Analysis of images acquired from coronal sectioning of the mouse brains showed that unilateral cannulae were correctly placed in the MePD for seven of nine mice (red dots in Figure 1A; *n* = 7). Analysis of images acquired from coronal sectioning of the mouse brains showed that bilateral cannulae were correctly placed in the MePD for 8 of 12 mice (red dots in Figure 1B; *n* = 8). A representative example of a coronal brain section from one mouse with a unilateral cannula implant is shown in Figure 1 aligned to the equivalent mouse brain stereotaxic coordinates.^33^ Mice with incorrect unilateral and bilateral cannulae placement were included for the purpose of validating the extent of spread of the infusions. Two mice with an incorrectly positioned unilateral cannula did not exhibit a change in LH pulse interval time following senktide administration (3 pmol, pre‐treatment: 17.50 ± 2.50 min, treatment: 18.75 ± 1.25 min; 0.3 pmol, pre‐treatment: 16.88 ± 3.13 min, treatment: 20 ± 5 min; 0.03 pmol, pre‐treatment: 18.75 ± 3.75 min, treatment: 19.17 ± 0.83 min; *n* = 2). SB222200 administration failed to block the suppressive effect of TMT on LH pulses in the three mice with incorrectly positioned bilateral cannula (SB222200 + TMT, pre‐treatment: 18.33 ± 1.67 min, treatment: 45 ± 2.88 min; SB222200 only, pre‐treatment: 19.17 ± 0.83 min, treatment: 20.83 ± 4.17 min; *n* = 3).

### 3.2 Administration of senktide into the MePD dose‐dependently inhibits pulsatile LH secretion in adult OVX mice

**FIGURE 2:**
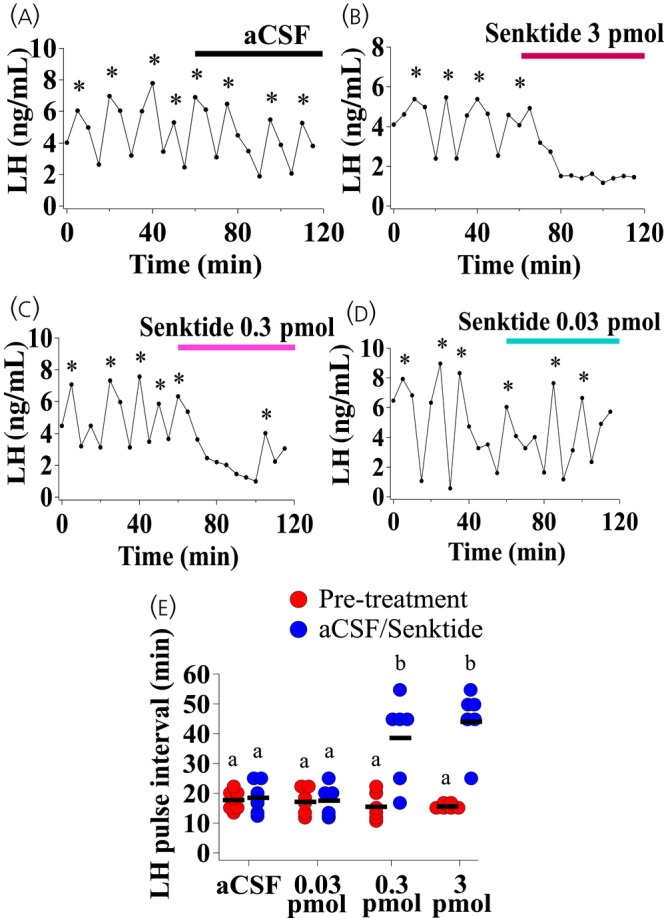
Dose‐dependent inhibition of luteinizing hormone (LH) pulsatility by unilateral intra‐posterodorsal medial amygdala (MePD) infusion of senktide, a NK3R agonist, in adult ovariectomised (OVX) C57Bl6/J female mice. Representative LH pulse profile showing response to intra‐MePD infusion of (A) artificial cerebrospinal fluid (aCSF), (B) 0.03 pmol, (C) 0.3 pmol or (D) 3 pmol senktide. (E) Summary of average LH pulse interval for the 1 h pre‐senktide control period (0–60 min) and 1 h senktide infusion period (60–120 min). LH pulses detected by the DynePeak algorithm are indicated with an asterisk located above each pulse on the representative LH pulse profiles. 0.3 pmol, b = +++*p* < .0001; *n* = 6; 3 pmol, b = +++*p* < .0001; *n* = 6–7 per group.

Unilateral senktide delivery into the right MePD dose‐dependently inhibited pulsatile LH secretion. Administration of aCSF had no effect on LH pulse interval (Figure 2A; *n* = 7). Treatment with 0.03 pmol senktide had no significant effect on LH pulse interval (Figure 2B; *n* = 6). However, mice treated with the two higher doses of senktide, 0.3 pmol and 3 pmol, exhibited robust inhibition of pulsatile LH secretion with significantly increased LH pulse interval compared to the pre‐infusion control periods (Figure 2C,D; 0.3 pmol, b = +++*p* < .0001; *n* = 6; 3 pmol, b = +++*p* < .0001; *n* = 6). The result obtained from the repeated measures two‐way ANOVA and Tukey post‐hoc statistical analysis is provided in full in Figure S1. These data are summarised in Figure 2E.

### 3.3 Antagonism of NK3R blocks TMT‐induced reduction of LH pulse frequency

**FIGURE 3:**
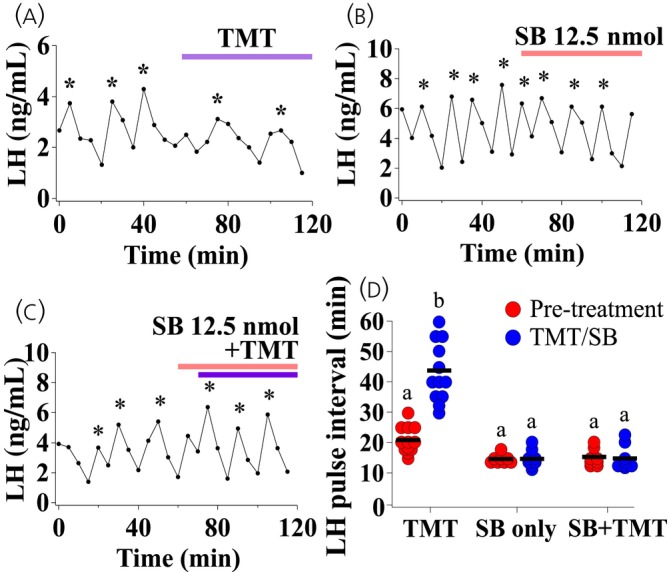
Acute 2,4,5‐trimethylthiazole (TMT)‐exposure suppresses luteinizing hormone (LH) pulsatility and bilateral intra‐posterodorsal medial amygdala (MePD) delivery of SB222200 (SB), an NK3R antagonist, blocked the effect of TMT on LH pulses in adult ovariectomised (OVX) C57Bl6/J female mice. Representative profile with (A) acute TMT exposure, (B) 12.5 nmol SB222200 infusion alone and (C) 12.5 nmol SB222200 infusion with TMT exposure. (D) Summary of the average LH pulse interval for the pre‐TMT control period (0–50 min), infusion of SB222200 (50–120 min) and TMT exposure (60–120 min). LH pulses detected by the DynePeak algorithm are indicated with an asterisk located above each pulse on the representative LH pulse profiles. Pre‐TMT versus TMT, b = +*p* < .05; TMT + SB222200, b = +++*p* < .0001; *n* = 8–12 per group.

Acute exposure to TMT increased LH pulse interval compared with the control period (Figure 3A; pre‐TMT vs. TMT, b = +*p* < .05; *n* = 12). Administration of SB222200 alone had no effect on LH pulsatility (Figure 3B; *n* = 8). Bilateral intra‐MePD administration of SB222200 blocked the TMT‐induced suppression of pulsatile LH secretion (Figure 3C,D; TMT + SB222200, b = +++*p* < .0001; *n* = 8). The result obtained from the repeated measures two‐way ANOVA and Tukey post‐hoc statistical analysis is provided in full in Figure S2. Results are summarised in Figure 3D.

### 3.4 In‐silico confirmation of the GnRH pulse generator responses to NK3R activation in the MePD and psychosocial stress exposure

**FIGURE 4:**
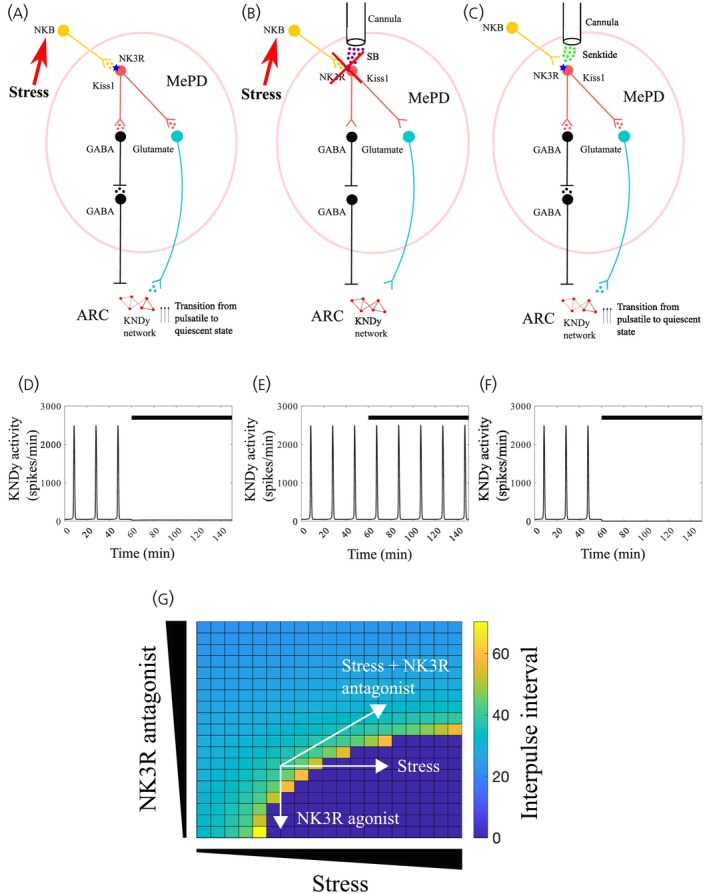
Proposed model of the interactions and pathways of the posterodorsal medial amygdala (MePD) neurocircuits involving stress/NK3R regulation over the hypothalamic GnRH pulse generator (ARC KNDy network) in ovariectomised (OVX) female mice. (A) According to our proposed model, stress activates NK3R signalling in MePD kiss1 neurons, which elevates their neuronal activity resulting in a net increase of stimulatory inputs from the MePD to the ARC KNDy network by activating two pathways: (i) a GABA‐GABA disinhibitory pathway that leads to a reduction in GABAergic tone arising from the MePD to the ARC KNDy network and (ii) a pathway involving glutamatergic MePD projection neurones. The stimulatory inputs from the MePD to the ARC KNDy network are enhanced leading to overstimulation of this network resulting in a transition from a pulsatile to a quiescent dynamic state of the GnRH pulse generator. (B) Intra‐MePD administration of SB222200 (SB), a specific NK3R antagonist, blocks the inhibitory effects of TMT on pulsatile LH secretion and (C) Intra‐MePD administration of senktide possibly activates NK3R on kiss1 neurons increasing their neuronal activity leading to overstimulation of the ARC KNDy network resulting in a transition from a pulsatile to a quiescent dynamic state of the GnRH pulse generator. To verify the LH pulse frequency response to predator odour exposure and modulation of NK3R signalling in the MePD, we build upon the existing model of the MePD kiss1, GABA, and glutamate neuronal circuitry, and incorporate NK3R expression in the MePD kiss1 neurons, where (D) corresponds to the system under stress, (E) corresponds to the system under stress and with SB administration, (F) corresponds to the system with senktide administration, and (G) presents a heatmap depicting the relationship between stress and NK3R antagonism on the LH interpulse interval.

To verify the LH pulse frequency response to psychosocial stress exposure and modulation of NK3R signalling in the MePD, we extended our mathematical model of the GnRH pulse generator, which incorporates inputs from the MePD neural circuitry comprised of kiss1, GABA and glutamate signalling.^8^, ^10^, ^38^ The existing model of the MePD circuitry involves MePD kiss1 neurons signalling to glutamatergic and GABA‐GABA disinhibitory projections extending from the MePD to the ARC KNDy network.^38^ The model we consider in the current study builds upon the existing model of the MePD kiss1, GABA and glutamate neuronal circuitry, and incorporates NK3R expression in the MePD kiss1 neurons (Figure 4A–C). The model successfully reproduces the inhibitory effect of psychosocial stress exposure and MePD NK3R activation on LH pulsatility (Figure 4A,C,D,F). Furthermore, blocking NK3R signalling in the MePD in the presence of predator odour has no effect on LH pulses (Figure 4B,E). The model assumes that predator odour is acting by increasing the activity of MePD kiss1 neurons via activation of the NK3R located on the kiss1 neurons in this region. The increase in MePD kiss1 neuronal activity possibly results in a net increase of stimulatory inputs to the ARC KNDy network. This results in an inhibition of ultradian oscillations, as the KNDy network undergoes a bifurcation and an upper threshold of input activity is crossed leading to termination of the pulsatile dynamics of this network (Figure 4D,F,G). Based on these assumptions the model verifies our experimental findings: (i) predator odour exposure inhibits LH pulses, (ii) activation of NK3R in the MePD inhibits LH pulses and (iii) NK3R antagonism in the MePD blocks stressor‐induced inhibition of LH pulse frequency.

## 4 DISCUSSION

In this study, we show that activation of NK3R in the MePD, via intra‐MePD administration of senktide, dose‐dependently inhibits GnRH pulse generator frequency in female OVX mice. Moreover, acute exposure to the predator odour, TMT, suppressed pulsatile LH secretion and this effect was completely blocked by NK3R antagonism with SB222200 in the MePD. These data demonstrate for the first time that psychosocial stress‐induced suppression of the GnRH pulse generator is mediated at least in part by the NK3R system in the MePD of female OVX mice.

In recent years, animal and human studies examining the effect of NK3R signalling on the function of the HPG axis have accumulated largely conflicting evidence. The first clinical study of NKB administration in healthy volunteers showed that intravenous infusion of NKB had no effect on reproductive hormone secretion.^39^ Although, NK3R antagonism has also been shown to decrease basal LH levels in humans.^40^, ^41^ Senktide injections stimulate LH release in castrated male macaques^42^ and gonadal intact ewes^43^ while central administration of NKB‐induced hypothalamic multiunit activity (MUA) volleys, an electrophysiological correlate of GnRH pulse generator activity, in the OVX goat.^44^ In contrast, ICV administration of senktide inhibits hypothalamic MUA volley frequency and dose‐dependently suppresses LH pulsatility irrespective of the steroidal milieu in female rats^23^, ^29^ and in OVX mice.^45^ On the other hand, studies have also shown that central administration of senktide increases serum LH levels in the presence of physiological levels of estradiol in rats^46^ and in female rats with arrested puberty due to chronic undernutrition senktide administration elicits LH release.^47^ The effect of NK3R activation on the function of the HPG axis, via central administration of senktide, appears to be dependent on the specific species under investigation and the sex steroid milieu while the site of action is unknown. Studies targeting the ARC showed that direct injections of senktide into this nucleus dose‐dependently suppressed LH pulses in OVX low‐level estradiol replaced rats.^48^ Recent experimental evidence and mathematical modelling have shed light on the modulatory effect of NKB and gonadal steroids on the level of excitability within the ARC KNDy network. The pulsatile behaviour of the KNDy network is determined by an upper and a lower bifurcation point, which is dependent on the basal activity and exogenous activation of the network.^8^ Gonadal steroids modulate the level of excitability within the ARC KNDy network, for example, across the ovarian cycle.^10^ This phenomenon is due to a sex steroid‐induced shift in the lower and upper bifurcation points of the system. The excitability of the network is partly modulated by the strength of NKB and dynorphin signalling as the levels of gonadal steroids fluctuate between the different phases of the reproductive cycle. Increases in NKB signalling strength in the presence of high oestrogen levels accelerate pulsatile LH secretion and, in the presence of low oestrogen, decelerate LH pulse frequency; thus, there is a strong negative correlation between changes in NKB signalling strength and changes in network excitability.^10^ Recently, it has been shown that selective activation of the NK3R signalling pathway in the MePD increases LH secretion in the presence of oestrogen in female kiss1 knock‐out mice.^28^ In contrast, we have shown in the present study that senktide‐induced activation of NK3R in the MePD dose‐dependently suppresses pulsatile LH secretion in OVX mice. The mechanism by which MePD NK3R signalling alters the activity of the GnRH pulse generator is unknown; however, it is likely its physiological relevance may be related to stress.

The MePD is known to be involved in coordinating stress cues with the neuroendocrine reproductive axis. Predator odour exposure, a classic rodent model of PTSD, robustly activates the MePD^18^ and suppresses pulsatile LH secretion,^3^ which concurs with psychosocial stress‐induced reduction in c‐fos expression in ARC kiss1 neurons.^11^ Recently, we have also shown that signalling through the corticotropin‐releasing factor (CRF) type 2 receptor (CRFR2)‐selective ligand urocortin 3 (Ucn3) in the MePD is involved in mediating the inhibitory effects of predator odour stress on LH pulsatility.^12^, ^14^, ^49^ Moreover, NKB and NK3R, which are expressed in the amygdala of various species including rodents,^26^ are implicated in processing stress and anxiety behaviours.^29^, ^30^, ^31^ Social isolation stress upregulates brain‐wide *Tac2* gene (encoding NKB) transcription.^50^ Furthermore, chemogenetic silencing of NKB‐expressing neurons in the central amygdala blocks distinct stress‐induced behavioural changes in mice.^50^ ICV administration of the NK3R antagonist, SB222200, blocks immunological stress‐induced suppression of LH pulses in OVX rats.^29^ In the present study, NK3R antagonism specific to the MePD blocks the inhibitory effect of TMT, a psychological stressor, on LH pulsatility in OVX female mice. The presence of estradiol is known to augment the suppressive effects of stress providing a sensitising effect on the stress‐induced inhibition of GnRH pulse generator activity in many species, from rodents to primates.^51^, ^52^, ^53^, ^54^ The present studies were performed in OVX mice, and the data presented support the conclusion that NK3R signalling is important for predator odour‐induced inhibition of LH pulsatility. These observations may differ if estradiol was present. The presence of estradiol may change the effects of stress on GnRH pulse generator activity mediated by MePD NK3R signalling, as in previous studies where NK3R agonist treatment was shown to increase LH release in response to fasting, a metabolic stressor, in pubertal rats.^47^ Oestrogen downregulates NK3R expression in the ARC while treatment with estradiol has been shown to increase the number of NK3R‐containing cell bodies in the MePD.^28^ Nevertheless, the present findings reveal a novel role for MePD NK3R signalling in integrating psychological stress signals with the GnRH pulse generator in the absence of circulating ovarian steroids in mice. These findings are clinically relevant as the potential future licencing of NK3R antagonists for menopausal hot flushes may also have anxiety and fear‐reducing effects. Pre‐clinical studies have shown that blocking *Tac* signalling in mice attenuates fear memory consolidation, which has potential as early therapeutics for anxiety and post‐trauma interventions to prevent post‐traumatic stress development.^55^ Moreover, administration of senktide into the amygdala of rats augmented fear‐potentiated startle, an effect blocked by NK3R antagonism, indicating that activation of NK3Rs in the amygdala of rats increases fear‐potentiated startle.^56^

NK3R‐expressing neurons in the MePD are surrounded by a dense NKB fibre network.^28^ Although the origin of these NKB fibres is not known they may arise from neighbouring amygdaloid nuclei, such as the central amygdala, which contain dense NKB neuronal populations.^57^ NK3R antagonism in the central amygdala modulates fear memory consolidation that occurs during a traumatic event^32^ and NK3R antagonism in the centromedial amygdala decreases fear memory consolidation in the male but increases fear memory consolidation in females in the pro‐estrus phase of the cycle.^58^ The central amygdala is also known to be involved in stress‐induced suppression of LH pulses.^3^ In the presence of psychological stress, the NKB system in the central amygdala may communicate with the NK3R system in the MePD to integrate the inhibitory effects of stress. Interestingly, ICV administration of senktide induces c‐fos expression in PVN CRF neurons,^59^ although CRF receptor antagonism does not prevent the senktide‐induced suppression of the GnRH pulse generator in OVX rats^29^; thus, it is unlikely that CRF neurons are recruited by NKB neurons to suppress the GnRH pulse generator, despite their ability to suppress pulsatile LH secretion upon activation.^60^ Gene expression profiles in the amygdala have identified and validated Ucn3 and Tacr3 genes to be involved in fear/anxiety traits in rats.^61^ Our previous findings show that MePD Ucn3 neurons mediate stress‐induced suppression of LH pulses,^12^ while in the current study, we show MePD NK3R signalling has a similar effect on pulsatile LH secretion in the presence of stress, thus both Ucn3 and NK3R neurons within the MePD play a role in mediating the suppressive effects of stress on the GnRH pulse generator. We have also shown that optogenetic stimulation of Ucn3 neurons in the MePD augments CORT secretion.^49^ Previous studies have shown that blockade of *Tac* receptors in the brain abolishes pain‐induced c‐fos expression in paraventricular corticotrophin‐releasing hormone neurones,^62^ while facilitating active stress‐coping strategies in the forced swim test,^63^ and both NK3R and Ucn3 neurons are highly expressed in the MePD.^28^, ^64^ This indicates that MePD NK3R activity may interact with the HPA axis in stressful situations. Although it was beyond the scope of this study to examine the effect of silencing NK3R signalling in the MePD on TMT‐induced CORT secretion, we cannot exclude the possibility that MePD Ucn3 neurons express NK3R or interact with NK3R expressing neurons in the MePD to modulate the HPG and possibly HPA axis activity. The potential interaction between these two systems in modulating the HPG and possibly the HPA axes remains to be explored.

Functional studies performed in rodents have demonstrated that kiss1 injections into the MePD led to an increase in LH secretion whereas blocking endogenous kiss1 signalling in this region decreases LH pulse frequency.^65^ Importantly, low‐frequency optogenetic activation of MePD kiss1 neurons increases the frequency of pulsatile LH secretion in OVX mice,^13^ indicating a stimulatory influence from these neurons to the ARC KNDy network. Interestingly, the MePD kiss1 signalling pathway has been recently identified to be independent from the MePD NK3R signalling pathway despite both being involved in stimulating the release of LH.^28^ NK3R signalling in the MePD augmented LH release in a female‐specific and oestrogen‐dependent manner, although the effect on the dynamics of pulsatile LH secretion was not explored.^28^ In the current study, the suppression of pulsatile LH secretion following the activation of MePD NK3R neurons in mice with functional kiss1 signalling and in the absence of circulating sex steroids, suggests the recruitment of the ARC KNDy network underlying GnRH pulse generation.

The discovery that activation of MePD kiss1 neurons increases the frequency of the GnRH pulse generator raised the hypothesis for the involvement of intranuclear GABA‐GABA disinhibitory interactions, typical of limbic pallidal structures, such as the MePD. Moreover, the MePD sends functional GABAergic projections to the ARC,^66^ providing a possible route via which MePD kiss1 neurons modulate the activity of the GnRH pulse generator. Neuropharmacological studies aiming to untangle this functional neural circuitry in the MePD have demonstrated that GABA and glutamate neurotransmission within this region is necessary for kiss1 neurons to exert their upstream control over the GnRH pulse generator.^38^ These experimental observations led to the development of a mathematical model to verify the hypothetical mechanism that low‐frequency activation of MePD kiss1 neurons may provide a direct stimulatory signal to downstream GABA interneurons, which, in turn, suppress inhibitory GABAergic projections from the MePD to ARC KNDy neurons resulting in an increase in GnRH pulse generator activity. Additionally, the activation of a glutamatergic projection from the MePD to the ARC was predicted to result in silencing of the KNDy network due to excessive activation, as the GnRH pulse generator is highly sensitive to incoming stimuli.^38^ This interpretation is in line with mathematical modelling of the ultradian oscillation of the ARC KNDy network, which is known to work on a bifurcation system that is terminated as an upper threshold of basal neuronal activity is reached.^8^, ^10^ Basal activity within the ARC KNDy network may reflect endogenous activity or mimic inputs that the network can receive externally from other brain regions, such as the MePD.

In the present study, we build upon our existing model of the neuronal circuitry in the MePD controlling GnRH pulse generator activity and introduce the hypothetical presence of NK3R on the MePD kiss1 neurons (see schematic Figure 4). Our extended mathematical model verifies the experimental observations that activation of MePD NK3R possibly located on kiss1 neurons may robustly stimulate kiss1 signalling in this region resulting in the suppression of GABAergic projections while also potentially increasing efferent glutamatergic signalling from the MePD to the ARC KNDy network (Figure 4C,F,G). This enhanced stimulatory input from the MePD increases ARC KNDy network excitability resulting in the transition from a pulsatile to a quiescent dynamic state of the GnRH pulse generator in the absence of gonadal steroids.^10^, ^38^ In our model, exposure to psychosocial stress is proposed to activate NK3R signalling in the MePD, exerting a similar effect on the activity of the GnRH pulse generator as senktide administration in the MePD (see Figure 4A,D,G). On the other hand, blocking MePD NK3R signalling in the presence of stress fails to activate MePD kiss1 signalling and the GnRH pulse generator remains in a pulsatile state (Figure 4B,E,G). Currently, the involvement of kiss1 signalling in modulating stress and anxiety‐like behaviour is unclear. Previous observations have shown that gonadectomised kiss1 receptor knockout male mice spend less time in the closed arms of the elevated plus maze and kiss1 receptor knockout intact male mice spent more time in the open arms compared with controls, indicating reduced anxiety‐like behaviour.^67^ On the other hand, selective activation of MePD kiss1 neurons increases the time spent and the number of entries into the open arms of the elevated plus maze, which suggest a significant reduction in the anxiety‐like behaviour of intact male mice.^68^ Since, in our model, NK3R‐induced kiss1 activation in the MePD results in the suppression of pulsatile LH secretion, like the effect of TMT exposure, a stressor, on LH pulses and NK3R antagonism blocks the inhibitory effect of TMT on LH pulses, which occurs by preventing MePD kiss1 activation, this suggests that activation of MePD kiss1 signalling may be anxiety‐inducing in OVX mice exposed to stress.

Interestingly, antagonism of NK3R signalling in the MePD with SB222200 did not affect pulsatile LH secretion in our OVX mice. This is an important observation for the proposed mechanism. This observation indicates that under non‐stress conditions MePD NKB neurons are relatively quiescent, thus solely pharmacologically blocking NK3R on kiss1 neurons without a corresponding increase in MePD NKB and kiss1 activity would make little difference to the net influence of the MePD over the KNDy system. However, it is possible that NK3R may be expressed on a separate population of neurons independent of MePD kiss1 neurons. In the neighbouring centromedial amygdala, NK3R is colocalised with GAD‐65,^58^ and in the medial pre‐optic area, RNAscope analysis revealed that *Tacr3*, NK3R, and mRNA are co‐expressed with vesicular GABA transporter mRNA.^69^ NK3R neurons could be GABAergic in the amygdala along with other regions. Thus, we cannot exclude the possibility of a more straightforward route where the stress‐activated NK3R is expressed on the GABAergic projection neurons, which may send direct efferents from the MePD to the KNDy network, decreasing GnRH pulse generator frequency.

Our findings show for the first time that NKB/NK3R signalling in the MePD mediates the inhibitory effect of TMT, a psychosocial stressor, on GnRH pulse generator frequency in the absence of ovarian steroids. How the NKB/NK3R neuronal populations in the MePD communicate with the ARC KNDy network to reduce GnRH pulse generator frequency remains to be established. Investigating the involvement of NKB/NK3R stress signalling in the MePD has furthered our understanding of the neural mechanisms engaged to mediate the inhibitory effects of stress on the reproductive system. These findings can ultimately contribute to the improvement of our treatment options for stress‐related reproductive disorders and have translational relevance to the ongoing development of NK3R antagonists for menopausal hot flushes.

## AUTHOR CONTRIBUTIONS

**Deyana Ivanova:** Conceptualization; data curation; formal analysis; investigation; methodology; validation; visualization; writing – original draft. **Margaritis Voliotis:** Formal analysis; investigation; software; validation; visualization. **Krasimira Tsaneva‐Atanasova:** Formal analysis; investigation; software; supervision; validation. **Kevin T. O'Byrne:** Conceptualization; funding acquisition; investigation; methodology; resources; supervision; validation; writing – review and editing. **Xiao‐Feng Li:** Conceptualization; funding acquisition; investigation; methodology; project administration; resources; supervision; validation; visualization.

## FUNDING INFORMATION

This study was funded by UKRI: BBSRC (BB/S000550/1; BB/W005913/1) and MRC (MR/N022637/1). Deyana Ivanova is a PhD student funded by MRC‐DTP studentship.

## CONFLICT OF INTEREST STATEMENT

The authors declare no conflicts of interest.

### PEER REVIEW

The peer review history for this article is available at https://www.webofscience.com/api/gateway/wos/peer-review/10.1111/jne.13384.

## Supporting information


**Figure S1.** Dose‐dependent inhibition of luteinizing hormone (LH) pulsatility by unilateral intra‐posterodorsal medial amygdala (MePD) infusion of senktide, a NK3R agonist, in adult ovariectomised (OVX) C57Bl6/J female mice. (A) Table showing the mean values for each group, (B) Table showing the Tukey and NeumanKeuls post hoc test result and (C) Table showing full result from 2‐way RM ANOVA.

**Figure S2.** Acute 2,4,5‐Trimethylthiazole (TMT)‐exposure suppresses luteinizing hormone (LH) pulsatility and bilateral intraposterodorsal medial amygdala (MePD) delivery of SB222200 (SB), a NK3R antagonist, blocked the effect of TMT on LH pulses in adult ovariectomised (OVX) C57Bl6/J female mice. (A) Table showing the mean values for each group, (B) Table showing the Tukey and NeumanKeuls post hoc test result and (C) Table showing full result from 2‐way.

## Data Availability

The data that support the findings of this study are available from the corresponding author upon reasonable request.
