# Supplementary material for: NK3R signalling in the posterodorsal medial amygdala is involved in stress‐induced suppression of pulsatile LH secretion in female mice
Source: J Neuroendocrinol. 2024 Mar 22;36(5):e13384. doi: 10.1111/jne.13384 (PMC11411622; doi:10.1111/jne.13384)
Supplement: Supplementary file 1 — Figure S1. Dose‐dependent inhibition of luteinizing hormone (LH) pulsatility by unilateral intra‐posterodorsal medial amygdala (MePD) infusion of senktide, a NK3R agonist, in adult ovariectomised (OVX) C57Bl6/J female mice. (A) Table showing the mean values for each group, (B) Table showing the Tukey and NeumanKeuls post hoc test result and (C) Table showing full result from 2‐way RM ANOVA. [file JNE-36-e13384-s001.pdf]

Supplementary Figure 1 a. aCSF/Senktide MePD

a	b	c	d	e	f	g	h
**Pre- aCSF group**	**aCSF group**	**Pre- 0.03 pmol group**	**0.03 pmol group**	**Pre- 0.3 pmol group**	**0.3 pmol group**	**Pre- 3 pmol group**	**3 pmol group**
**12.5**	**16.6667**	18.333333	13.333333	11	55	16.666667	45
15	25	13.333333	12.5	**13.75**	**45**	15	50
15	20	**16.666667**	**20**	11.6666667	16.6666667	15	25
20	12.5	11.666667	11.666667	20	45	**16.666667**	**55**
20	25	22.5	20	15	25	15	45
22.5	13.3333	22.5	25	22.5	45	15	50
18.3333	17.5						

Supplementary Figure 1 b. aCSF/Senktide MePD
StatsTukeyTest

Pair (x_vs_y)	DIFF	SE	q	q.05_39_6	Concl.T	**p-value**		q.5_39_p	Concl.NK
3 pmol group (h)_vs_Pre-3 pmol group (g)	28.611111	2.89697511	9.87620187	4.50909822	0	**4.15E-07**	8	4.50909822	0
3 pmol group (h)_vs_Pre-0.3 pmol group (e)	28.5833389	2.89697511	9.86661528	4.50909822	0	**4.25E-07**	7	4.37776248	0
3 pmol group (h)_vs_0.03 pmol group (d)	27.0833333	2.89697511	9.34883191	4.50909822	0	**1.41E-06**	6	4.22177938	0
3 pmol group (h)_vs_Pre-0.03 pmol group (c)	26.9444445	2.89697511	9.3008892	4.50909822	0	**1.57E-06**	5	4.03023211	0
3 pmol group (h)_vs_Pre-aCSF group (a)	26.4285762	2.79159507	9.4671955	4.50909822	0	**1.07E-06**	4	3.78296082	0
3 pmol group (h)_vs_aCSF group (b)	25.5952381	2.79159507	9.16867865	4.50909822	0	**2.14E-06**	3	3.43582313	0
3 pmol group (h)_vs_0.3 pmol group (f)	5.55555555	2.89697511	1.91770911	4.50909822	1	0.87140314	2	2.85399888	1
0.3 pmol group (f)_vs_Pre-3 pmol group (g)	23.0555555	2.89697511	7.95849277	4.50909822	0	**3.53E-05**	7	4.37776248	0
0.3 pmol group (f)_vs_Pre-0.3 pmol group (e)	23.0277833	2.89697511	7.94890617	4.50909822	0	**3.61E-05**	6	4.22177938	0
0.3 pmol group (f)_vs_0.03 pmol group (d)	21.5277778	2.89697511	7.4311228	4.50909822	0	**0.00011799**	5	4.03023211	0
0.3 pmol group (f)_vs_Pre-0.03 pmol group (c)	21.388889	2.89697511	7.38318009	4.50909822	0	**0.00013156**	4	3.78296082	0
0.3 pmol group (f)_vs_Pre-aCSF group (a)	20.8730207	2.79159507	7.4770947	4.50909822	0	**0.00010627**	3	3.43582313	0
0.3 pmol group (f)_vs_aCSF group (b)	20.0396825	2.79159507	7.17857786	4.50909822	0	**0.000209**	2	2.85399888	0
aCSF group (b)_vs_Pre-3 pmol group (g)	3.0158729	2.79159507	1.08034039	4.50909822	1	0.99410263	6	4.22177938	1
aCSF group (b)_vs_Pre-0.3 pmol group (e)	2.98810079	2.79159507	1.07039191	4.50909822	1	0.9944251	5	4.03023211	1
aCSF group (b)_vs_0.03 pmol group (d)	1.48809524	2.79159507	0.53306271	4.50909822	1	0.99993978	4	3.78296082	1
aCSF group (b)_vs_Pre-0.03 pmol group (c)	1.3492064	2.79159507	0.48331021	4.50909822	1	0.99996902	3	3.43582313	1
aCSF group (b)_vs_Pre-aCSF group (a)	0.83333814	2.68207778	0.31070618	4.50909822	1	0.99999851	2	2.85399888	1
Pre- aCSF group (a)_vs_Pre-3 pmol group (g)	2.18253476	2.79159507	0.78182355	4.50909822	1	0.99923237	5	4.03023211	1
Pre- aCSF group (a)_vs_Pre-0.3 pmol group (e)	2.15476265	2.79159507	0.77187507	4.50909822	1	0.99929372	4	3.78296082	1
Pre- aCSF group (a)_vs_0.03 pmol group (d)	0.6547571	2.79159507	0.23454587	4.50909822	1	0.99999979	3	3.43582313	1
Pre- aCSF group (a)_vs_Pre-0.03 pmol group (c)	0.51586826	2.79159507	0.18479337	4.50909822	1	0.99999996	2	2.85399888	1
Pre- aCSF group (a)_vs_Pre-3 pmol group (g)	1.6666665	2.89697511	0.57531268	4.50909822	1	0.99989928	4	3.78296082	1
Pre- 0.03 pmol group (c)_vs_Pre-0.3 pmol group (e)	1.63889438	2.89697511	0.56572608	4.50909822	1	0.99991005	3	3.43582313	1
Pre- 0.03 pmol group (c)_vs_0.03 pmol group (d)	0.13888883	2.89697511	0.04794271	4.50909822	1	1	2	2.85399888	1
0.03 pmol group (d)_vs_Pre-3 pmol group (g)	1.52777767	2.89697511	0.52736997	4.50909822	1	0.999944	3	3.43582313	1
0.03 pmol group (d)_vs_Pre-0.3 pmol group (e)	1.50000555	2.89697511	0.51778338	4.50909822	1	0.99995054	2	2.85399888	1
Pre- 0.3 pmol group (e)_vs_Pre-3 pmol group (g)	0.02777212	2.89697511	0.00958659	4.50909822	1	1	2	2.85399888	1

KEY (figure 1b):

Pair = x group_vs_y group

DIFF = mean difference between groups

SE = standard error

q = the critical value from the Studentized Range Distribution

Concl.T = conclusion of Tukey test

Concl.NK = conclusion of Neuman-Keuls test

Supplementary Figure 1 c. aCSF/Senktide MePD
RM Two-way ANOVA

	Df	Sum Sq	Mean Sq	F value	Pr(>F)
Group	1	634.6	634.6	11.335	**0.00199++**
Treatment	3	506.4	168.8	3.015	**0.04428+**
Group:Treatr	3	692.2	230.7	4.121	**0.01403+**
Residuals	32	1791.6	56		

Supplementary Figure 1. Dose-dependent inhibition of luteinizing hormone (LH) pulsatility by unilateral intra-posterodorsal medial amygdala (MePD) infusion of senktide, a NK3R agonist, in adult ovariectomised (OVX) C57Bl6/J female mice. (A) Table showing the mean values for each group, (B) Table showing the Tukey and Neuman-Keuls post hoc test result and (C) Table showing full result from 2-way RM ANOVA.
