# Supplementary material for: NK3R signalling in the posterodorsal medial amygdala is involved in stress‐induced suppression of pulsatile LH secretion in female mice
Source: J Neuroendocrinol. 2024 Mar 22;36(5):e13384. doi: 10.1111/jne.13384 (PMC11411622; doi:10.1111/jne.13384)
Supplement: Supplementary file 2 — Figure S2. Acute 2,4,5‐Trimethylthiazole (TMT)‐exposure suppresses luteinizing hormone (LH) pulsatility and bilateral intraposterodorsal medial amygdala (MePD) delivery of SB222200 (SB), a NK3R antagonist, blocked the effect of TMT on LH pulses in adult ovariectomised (OVX) C57Bl6/J female mice. (A) Table showing the mean values for each group, (B) Table showing the Tukey and NeumanKeuls post hoc test result and (C) Table showing full result from 2‐way. [file JNE-36-e13384-s002.pdf]

Supplementary Figure 2 a. TMT/SB222200 MePD

a	b	c	d	e	f
**Pre- TMT group**	**TMT group**	**Pre- SB only group**	**SB only group**	**Pre- SB+TMT group**	**SB+TMT group**
18.3333333	30	13.75	15	12.5	12.5
25	55	**13.333333**	**13**	15	13.333333
25	40	15	11.25	**18.333333**	**15**
20	40	17.5	20	15	22.5
30	45	13.333333	13.333333	12.5	12.5
15	55	13.333333	17.5	13.75	11.666667
16.6666667	35	15	15	13.75	12.5
**17.5**	**32.5**	15	12.5	20	20
20	35				
20	40				
25	60				
17.5	50				

Supplementary Figure 2 b. TMT/SB222200 MePD
StatsTukeyTest

Pair (x_vs_y)	DIFF	SE	q	q.05_39_6	Concl.T	**p-value**		q.5_39_p	Concl.NK
TMT group (b)_vs_Pre-SB only group (c)	29.9131946	1.6016538	18.6764421	4.2128353	0	**8.51E-13**	6	4.2128353	0
TMT group (b)_vs_SB only group (d)	29.7048612	1.6016538	18.5463682	4.2128353	0	**8.56E-13**	5	4.02217152	0
TMT group (b)_vs_SB+TMT group (f)	29.4444444	1.6016538	18.3837758	4.2128353	0	**8.63E-13**	4	3.77595825	0
TMT group (b)_vs_Pre-SB+TMT group (e)	29.3402778	1.6016538	18.3187389	4.2128353	0	**8.67E-13**	3	3.43014966	0
TMT group (b)_vs_Pre-TMT group (a)	24.8148148	1.55383243	15.9700714	4.2128353	0	**1.80E-12**	2	2.85016051	0
Pre- TMT group (a)_vs_Pre-SB only group (c)	5.09837975	1.6016538	3.18319711	4.2128353	1	0.23612925	5	4.02217152	1
Pre- TMT group (a)_vs_SB only group (d)	4.89004638	1.6016538	3.05312319	4.2128353	1	0.27741964	4	3.77595825	1
Pre- TMT group (a)_vs_f	4.62962963	1.6016538	2.89053079	4.2128353	1	0.33507567	3	3.43014966	1
Pre- TMT group (a)_vs_Pre-SB+TMT group (e)	4.525463	1.6016538	2.82549387	4.2128353	1	0.35990282	2	2.85016051	1
Pre-SB+TMT group (e)_vs_Pre-SB only group (c)	0.57291675	1.64808817	0.34762506	4.2128353	1	0.9998685	4	3.77595825	1
Pre-SB+TMT group (e)_vs_SB only group (d)	0.36458338	1.64808817	0.22121594	4.2128353	1	0.99998597	3	3.43014966	1
Pre-SB+TMT group (e)_vs_SB+TMT group (f)	0.10416662	1.64808817	0.06320452	4.2128353	1	0.99999997	2	2.85016051	1
SB+TMT group (f)_vs_Pre-SB only group (c)	0.46875013	1.64808817	0.28442054	4.2128353	1	0.99995119	3	3.43014966	1
SB+TMT group (f)_vs_SB only group (d)	0.26041675	1.64808817	0.15801142	4.2128353	1	0.99999737	2	2.85016051	1
SB only group (d)_vs_Pre-SB only group (c)	0.20833338	1.64808817	0.12640912	4.2128353	1	0.99999914	2	2.85016051	1

KEY (figure 2b):

Pair = x group_vs_y group

DIFF = mean difference between groups

SE = standard error

q = the critical value from the Studentized Range Distribution

Concl.T = conclusion of Tukey test

Concl.NK = conclusion of Neuman-Keuls test

Supplementary Figure 2 c. TMT/SB222200 MePD
RM Two-way ANOVA

	Df	Sum Sq	Mean Sq	F value	Pr(>F)
Group	1	92.1	92.06	3.05	**0.0487+**
Treatment	2	714.9	238.31	9.707	**0.0000407+++**
Group:Treatr	2	228.3	76.12	3.1	**0.0353+**
Residuals	48	1178.5	24.55		

Supplementary Figure 2. Acute 2,4,5-Trimethylthiazole (TMT)-exposure suppresses luteinizing hormone (LH) pulsatility and bilateral intra-posterodorsal medial amygdala (MePD) delivery of SB222200 (SB), a NK3R antagonist, blocked the effect of TMT on LH pulses in adult ovariectomised (OVX) C57Bl6/J female mice. (A) Table showing the mean values for each group, (B) Table showing the Tukey and Neuman-Keuls post hoc test result and (C) Table showing full result from 2-way
